# *Salmonella* Salamae and *S.* Waycross isolated from Nile perch in Lake Victoria show limited human pathogenic potential

**DOI:** 10.1038/s41598-022-08200-5

**Published:** 2022-03-10

**Authors:** Yaovi Mahuton Gildas Hounmanou, Zebedayo Baniga, Vanesa García, Anders Dalsgaard

**Affiliations:** 1 Department of Veterinary and Animal Sciences, University of Copenhagen, Frederiksberg, Denmark; 2 Research Unit of Applied Microbiology and Pharmacology of Natural Substances, University of Abomey-Calavi, Godomey, Benin; 3 Department of Veterinary Medicine and Public Health, Sokoine University of Agriculture, Morogoro, Tanzania; 4 Laboratorio de Referencia de Escherichia coli (LREC), Departamento de Microbioloxía e Parasitoloxía, Facultade de Veterinaria, Instituto de Investigación Sanitaria de Santiago de Compostela (IDIS), Universidade de Santiago de Compostela (USC), 27002 Lugo, Spain

**Keywords:** Computational biology and bioinformatics, Microbiology, Pathogenesis

## Abstract

Non-*enterica* subspecies of *Salmonella enterica* are rarely associated with human infections. Paradoxically, food safety legislations consider the entire genus *Salmonella* as pathogenic to humans. Globally, large amounts of seafoods are rejected and wasted due to findings of *Salmonella*. To inform better food safety decisions, we investigated the pathogenicity of *Salmonella* Salamae 42:r- and *Salmonella* Waycross isolated from Nile perch from Lake Victoria. Genome-wide analysis revealed absence of significant virulence determinants including on key *Salmonella* pathogenicity islands in both serovars. In epithelial cells, *S.* Salamae showed a weak invasion ability that was lower than the *invH* mutant of *S.* Typhimiurium used as negative control. Similarly, *S*. Salamae could not replicate inside macrophages. Moreover, intracellular replication in *S.* Waycross strains was significantly lower compared to the wild type *S.* Typhimurium. Our findings suggest a low pathogenicity of *S*. Salamae reinforcing the existing literature that non-*enterica* subspecies are avirulent. We propose that food legislations and actions taken on findings of *Salmonella* are revisited to avoid wasting valuable sea- and other foods.

## Introduction

The genus *Salmonella* is classified into two species, *Salmonella enterica* and *Salmonella bongori*. *S. enterica,* contains over 2600 serovars and is subdivided into six subspecies which are *enterica* I, *salamae* II, *arizonae* IIIa, *diarizonae* IIIb, *houtenae* IV, and *indica* VI^1^. Serovars belonging to subspecies *S. enterica*, which are around 1600, are mainly non-typhoid *Salmonella* associated with gastroenteritis and salmonellosis in humans and animals^1,2^. The hosts of *S. enterica* subsp. *enterica* are warm-blooded animals and their occurrence in aquatic environments is often associated with fecal pollution due to influx of fecal wastes from humans and animals^3–5^. In contrast, the hosts of the *Salmonella* non-*enterica* subspecies are commonly cold-blooded animals such as reptiles and amphibians, and they rarely cause infection in animals and humans^6–8^.

Although *Salmonella enterica* and non-*enterica* subspecies can be isolated from many sources, it is the serovars of subspecies *enterica* hosted and transmitted by terrestrial animals that constitute the lead causes of salmonellosis in humans^9^. The relative contribution of seafood including fish to the global epidemiology of human salmonellosis is very low and mostly limited to *S.* Weltevreden in Asia; a serovar which is also an enterica subspecies^9,10^. In a recent study, we identified *S.* Waycross of subspecies *enterica* and the serovar 42:r- of subspecies Salamae from Nile perch (*Lates niloticus*) and water sampled far off the shores of Lake Victoria containing low levels of fecal contamination^11^.

In East Africa, the Nile perch industry has suffered economic losses due to failure to comply with the microbiological standards of the European Union (EU), the main export market for the Lake Victoria’s fisheries. Through the Rapid Alert System for Food and Feed (RASFF) of the EU (https://ec.europa.eu/food/safety/rasff_en), imports of fresh and frozen Nile perch products from Lake Victoria have often been suspended due to findings of *Salmonella* spp. which has led to rejections or notification of consignments and severe economic losses^11,12^. Since the existing food safety legislation in the EU and elsewhere is based on identification of *Salmonella* at the genus level and actions are taken irrespective of which sub-species or serovar is found, fish products containing *S*. Waycross and *S*. Salamae would be rejected by food safety authorities in the importing countries. Such actions are taken despite that the public health importance of these and other non*-enterica Salmonella* serovars is still uncertain.

Due to their rare involvement in human and animal infections, it has been proposed to consider the non-*enterica Salmonella* subspecies like *S*. Salamae, as opportunistic pathogens^13^. Moreover, *S.* Waycross, although a member of the *enterica* subspecies, is not commonly reported associated with human infection^14,15^. From a food safety and public health point of view, it is therefore important to elucidate the full pathogenic potential of non*-enterica* subspecies but also rare serovars of the *enterica* subspecies to inform better food safety decisions without unnecessary losses of safe foods and sale. Knowledge about non-*enterica* subspecies is still relatively limited but studies carried out reveal that their virulence and capacity to colonize humans is very limited^13^. Few genomic studies have been conducted on non-*enterica* subspecies and little is known about the differences in for instance the *Salmonella* pathogenicity islands between *enterica* and non-*enterica* subspecies^16^. Using whole genome sequence analysis coupled with epithelial and macrophage cell lines infection, we reveal in this report a potentially poor pathogenicity of *Salmonella* Salamae and *S*. Waycross isolated from Nile perch and water obtained in the Tanzanian basin of Lake Victoria.

## Results

### Genetic profiles of *S*. Waycross and *S*. Salamae

The genome data confirmed the initial serotyping result that isolates were *S*. *enterica* subsp*.* Salamae serovar 42:r- and *S*. *enterica* subsp. *enterica* serovar Waycross (Table 1). All *S*. Salamae 42:r- were assigned to sequence type (ST) 1208, while *S.* Waycross were ST2460 (five strains) and ST3691 (two strains) (Table 1).

**Table 1:** Genomic characteristics of *S.* Salamae 42:r:- and *S.* Waycross.

Sample number	A7	A8	A9	B6	D5	D6	I1	A1	C2	D3	E5	E9	G5	H6
Sample type	Fish intestines	Fish intestines	Fish intestines	Fish intestines	Lake water	Lake water	Fish intestines	Fish surface	Fish intestines	Fish intestines	Fish intestines	Fish intestines	Fish intestines	Lake water
# Contigs	150	118	172	148	94	123	79	116	67	82	70	83	78	93
Genome size (bp)	4,775,198	4,792,107	4,797,164	4,763,962	4,800,001	4,788,828	4,799,696	4,737,395	4,742,170	4,742,542	4,741,533	4,741,231	4,711,795	4,700,978
Coverage	52	64	46	62	91	56	86	42	61	79	86	79	70	71
Accession No	SAMEA5927480	SAMEA5927481	SAMEA5927482	SAMEA5927483	SAMEA5927486	SAMEA5927487	SAMEA5927492	SAMEA5927479	SAMEA5927484	SAMEA5927485	SAMEA5927488	SAMEA5927489	SAMEA5927490	SAMEA5927491
Subspecies	*II(Salamae)*	*II(Salamae)*	*II(Salamae)*	*II(Salamae)*	*II(Salamae)*	*II(Salamae)*	*II(Salamae)*	*enterica*	*enterica*	*enterica*	*enterica*	*enterica*	*enterica*	*enterica*
Serovars	42:r:-	42:r:-	42:r:-	42:r:-	42:r:-	42:r:-	42:r:-	Waycross	Waycross	Waycross	Waycross	Waycross	Waycross	Waycross
H1-phase-1 flagella	r	r	r	r	r	r	r	z4,z23	z4,z23	z4,z23	z4,z23	z4,z23	z4,z23	z4,z23
MLST	1208	1208	1208	1208	1208	1208	1208	2460	2460	2460	2460	2460	3691	3691
Resistance genotypes	parC:p.T57S; *aac(6')-Iaa*	parC:p.T57S; *aac(6')-Iaa*	parC:p.T57S; *aac(6')-Iaa*	parC:p.T57S; *aac(6')-Iaa*	parC:p.T57S; *aac(6')-Iaa*	parC:p.T57S; *aac(6')-Iaa*	parC:p.T57S; *aac(6')-Iaa*	parC:p.T57S; *aac(6')-Iaa*	parC:p.T57S; *aac(6')-Iaa*	parC:p.T57S; *aac(6')-Iaa*	parC:p.T57S; *aac(6')-Iaa*	parC:p.T57S; *aac(6')-Iaa*	parC:p.T57S; *aac(6')-Iaa*	parC:p.T57S; *aac(6')-Iaa*
Phenotypic resistance^a^	None	SMX	None	None	None	None	None	NAL; SMX	SMX	None	None	None	NAL; SMX	NAL;SMX

^a^NAL, nalidixic acid; SMX, sulfamethoxazole.

The antimicrobial resistance typing data revealed that the strains only harbored the *aac(6’)laa* gene encoding aminoglycoside resistance and a mutation in *parC* (p.T57S) encoding fluoroquinolone resistance. However, the previous phenotypic resistance data established by MIC indicated that five *S*. Salamae 42:r- and three *S*. Waycross strains were susceptible to all tested antimicrobials (Table 1). The remaining strains showed phenotypic resistance to sulfamethoxazole and nalidixic acid (Table 1). The genomes contained no plasmid replicons.

The *in-silico* analysis of the *S.* Salamae 42:r- genomes resulted in the prediction of six prophage regions in each strain (but seven for strain A9) (Table S1). The genome sizes of these prophage regions varied from 4.8 to 66.1 kb, with a GC content ranging from 48.0 to 53.7%. Of the total 43 predicted prophages, 21 were intact, whereas 17 were incomplete and the remaining five were questionable. The number of prophages differed between *S.* Salamae and *S.* Waycross as the latter showed more phage regions ranging from eight to 11 in each of the analyzed strains. The phages identified in *S.* Waycross varied in size from 6.1 to 41.8 kb and their GC content from 41.9 to 54.3%. Of the 69 predicted prophages in *S.* Waycross, 28 were intact, whereas 29 were incomplete and 12 were questionable. In *S.* Salamae 42:r-, the prophage regions identified showed similarity to known Enterobacteriaceae prophages where the predominant ones were sal3 (NC_031940, 32.6%) followed by HP2 (NC_003315, 17.8%), phiE255 (NC_009237), Gifsy1 (NC_010392) and SEN34 (NC_028699) with the three latter occurring at 16.4% proportions. Of the many prophage regions identified in the *S.* Waycross genomes, the most prevailing ones showed similarity to vB_SosS_Oslo (NC_018279, 15.9%), followed by Gifsy2 (NC_010393, 11.6%), and HP2 (NC_003315), sal3 (NC_031940) and BTP1 (NC_042346) with the three latter occurring in 10.1% proportions. Overall, *S.* Waycross genomes in contrast with *S.* Salamae harbored higher number and more diverse prophage regions as they seem to have experienced more phage insertions than *S.* Salamae.

### Pathogenicity determinants

**Figure 1:**
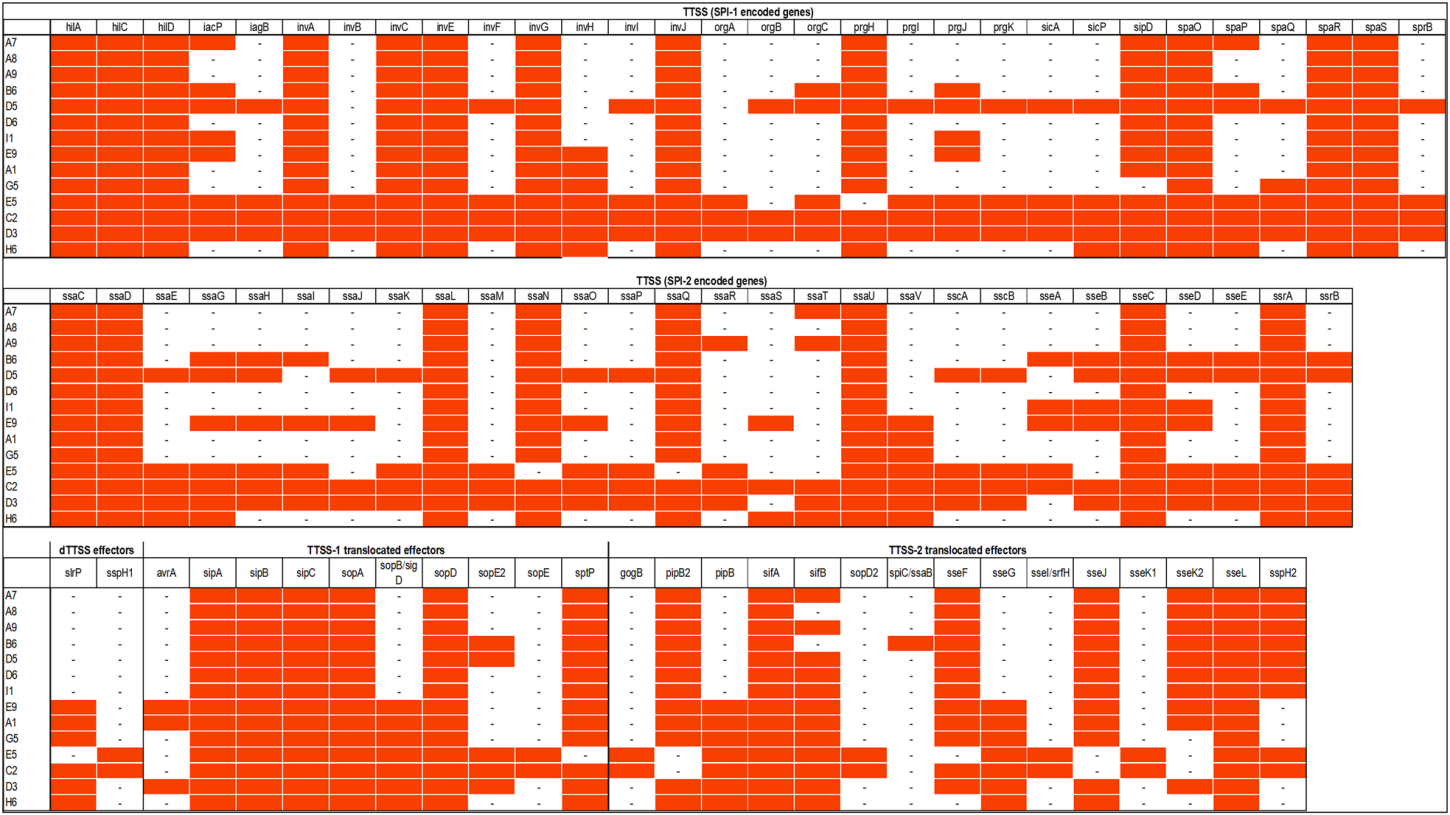
Presence/absence map of genes on SPI1 and SPI2 as well as their translocated effectors. The full red boxes represent presence of the indicated gene.

Virulence in *Salmonella* is mainly regulated by the SPIs (*Salmonella* Pathogenicity Islands) with SPI1 encoded genes involved in host cell invasion and SPI2 genes in survival of *Salmonella* in the host cell. Our *S*. Waycross and *S*. Salamae 42:r- strains, like most *Salmonella enterica* harbored SPI1 and SPI2 genes as well as their translocated effectors (Fig. 1). However, a number of significant deletions were observed in the two pathogenicity islands of the serovars. On SPI1, genes missing in most strains included *invB, invF, inv*H, *invI, orgA, orgB, orgC, prgI, prgJ prgK, sicA, sicP, spaP, spaQ,* and *sprB* (Fig. 1). On SPI2 important genes involved in survival in host cells including *ssaE, ssaG, ssaH, ssaI, ssaJ, ssaM, and ssaV* were absent in most of the *S*. Salamae 42:r- and *S*. Waycross strains. Clinically important type-three secretion system (T3SS) translocated effectors in *Salmonella* virulence such as *avrA, sopE, sopD, gogB*, and *sseK* were also absent in the two serovars (Fig. 1) despite lowering the detection threshold to 50%. The deletions on the pathogenicity islands occurred more in *S*. Salamae 42:r- than in *S*. Waycross as some *S*. Waycross strains showed full presence of the targeted genes, but both serovars completely lacked the *spv* locus with its five related genes (*spvA, B, C, D*, and *R*) indicating a low virulence potential of these serovars. Besides SPI1 and SPI2, both serovars contained SPI3, 4, 5 and 9 encoded genes.

### Genome-wide content comparison

**Figure 2:**
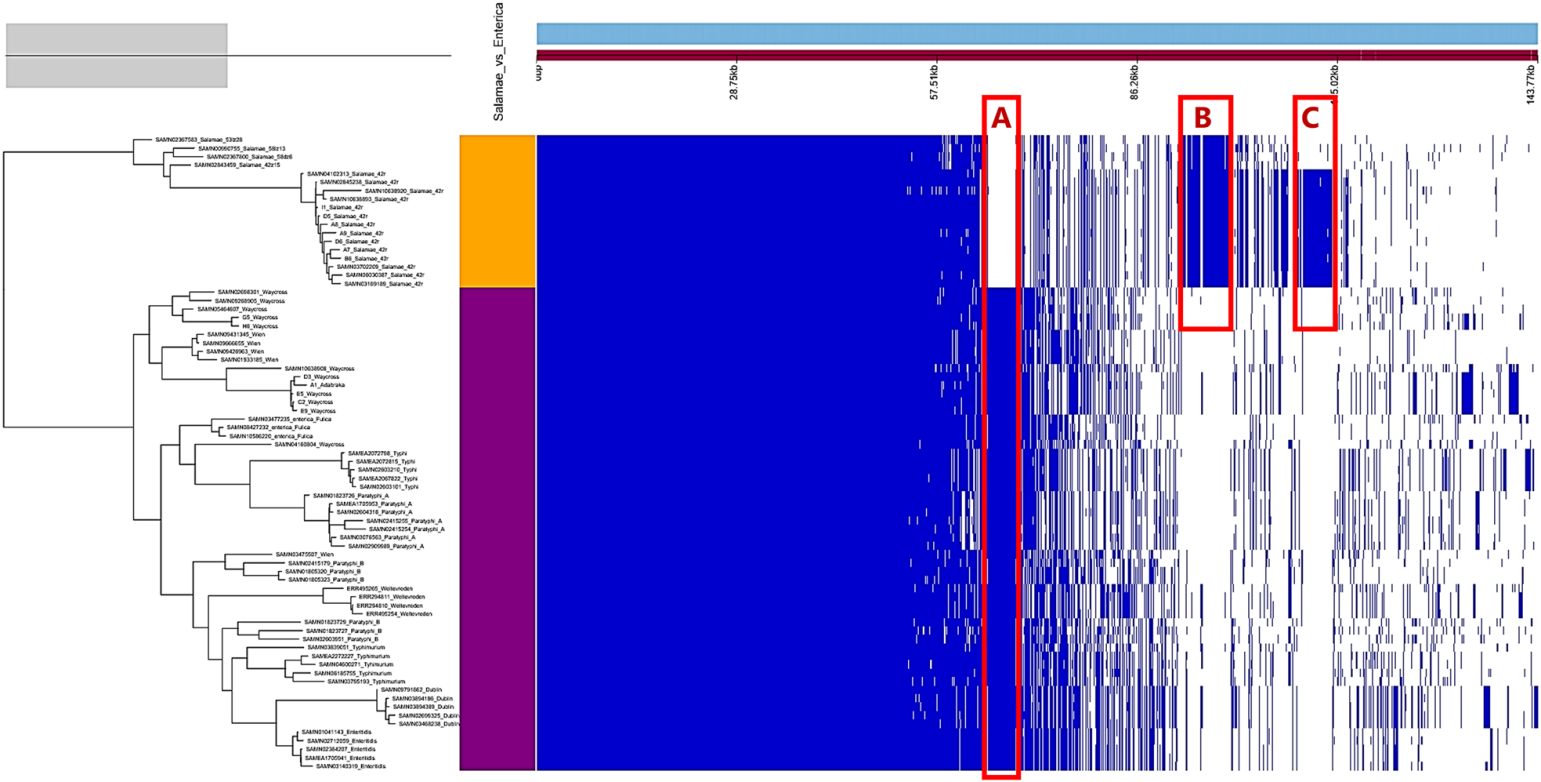
Pangenome view of 75 non-*enterica* and *enterica* subspecies of *Salmonella enterica.* All *S*. Salamae strains are in the clade colored orange while all subspecies *enterica* are colored purple. Unique coding sequences observed in the accessory genome are represented by the zones A, B and C.

The pan-genome analysis of 75 *enterica* and non-*enterica* subspecies of *Salmonella enterica* identified 14,438 total coding sequences (CDS), divided into 2,931 core CDS (shared by > 95% of strains), 38 soft core CDS, 2,984 shell CDS and 8,485 cloud CDS. The overall gene presence and absence data from the pangenome is provided in Table S2. In the accessory genome, the output gene presence/absence map (Fig. 2) revealed subspecies and/or serovar-based clustering with three characteristic unique regions in the genomes corresponding to the *enterica* subspecies including *S*. Waycross (Part A, Fig. 2), the *S*. Salamae subspecies (Part B) and the *S*. Salamae 42:r- serovar (Part C). These unique regions represent CDS specific to the subspecies and/or the serovars and were further analyzed for their potential role in virulence.

The unique CDS were retracted as described in Materials and Methods and mapped back to reference genomes of each of the three groups to identify their location and analyze their involvement in potential genetic evolution. This mapping revealed that the unique CDS did not correspond to one specific region in the genomes of *S*. Waycross and *S*. Salamae, but could rather be seen throughout the genomes with query covers between 81 and 100% across the entire genomes with variable GC contents indicating their potential external acquisition as a result of horizontal gene transfer (Fig. S1). This indicates that evolution/divergences at serovar level occurs not in a specific region of the genomes but across the genomes and should be further studied for their functional role in each targeted subspecies and / or serovar.

**Figure 3:**
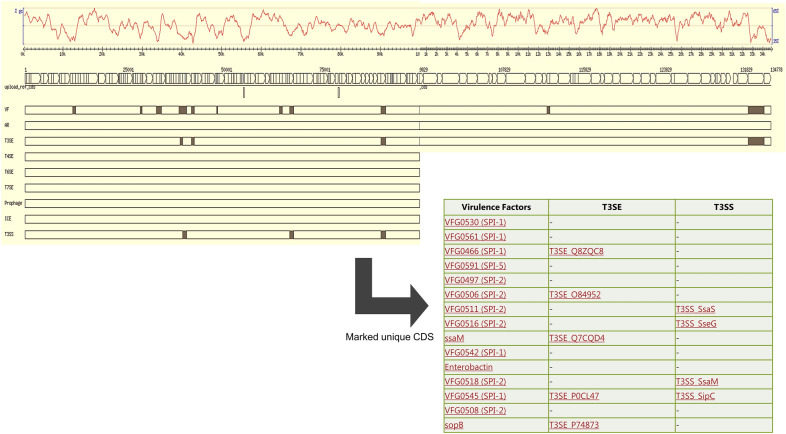
Annotated genes encoded in the unique CDS of the enterica species group (including *S.* Waycross) in Part A of Fig. 2. The solid marks in the upper image is displayed in the lower table with the actual genes names.

To provide understanding in the role of these regions specific to *S*. Waycross and *S.* Salamae 42:r-, the targeted CDS unique to these serovars i.e. Part A and Part C from the pangenome comparison were further analyzed. The results supports that *S.* Waycross clustering with other common pathogenic *enterica*-subspecies, contain genes encoding for virulence factors as well as type three secretion system genes and T3SS translocated effectors that are absent in *S*. Salamae 42:r- (Fig. 3). This reinforces that *S.* Waycross as an *enterica*-subspecies contains more pathogenicity markers compared to *S*. Salamae 42:r- despite the many deletions in its pathogenicity islands.

On the other hand, in the unique CDS of *S.* Salamae 42:r-, the genetic content shows gene clustering corresponding to two main prophage elements and the rest are hypothetical proteins (Fig. S2). We could not reconstruct any intact phage from these gene clusters. However, region #1 contains a number of phage particles such as repressors of immunity control, phage integrase, phage tail assembly proteins, endolysins and various phage hypothetical proteins (Fig. S2). Likewise, the second region of gene clusters specific to *S*. Salamae 42:r- contained phage components such as phage tail fiber assembly proteins, phage related transmembrane proteins, holin and Ner-like proteins. Overall, none of the components of these clusters point to virulence determinants.

**Figure 4:**
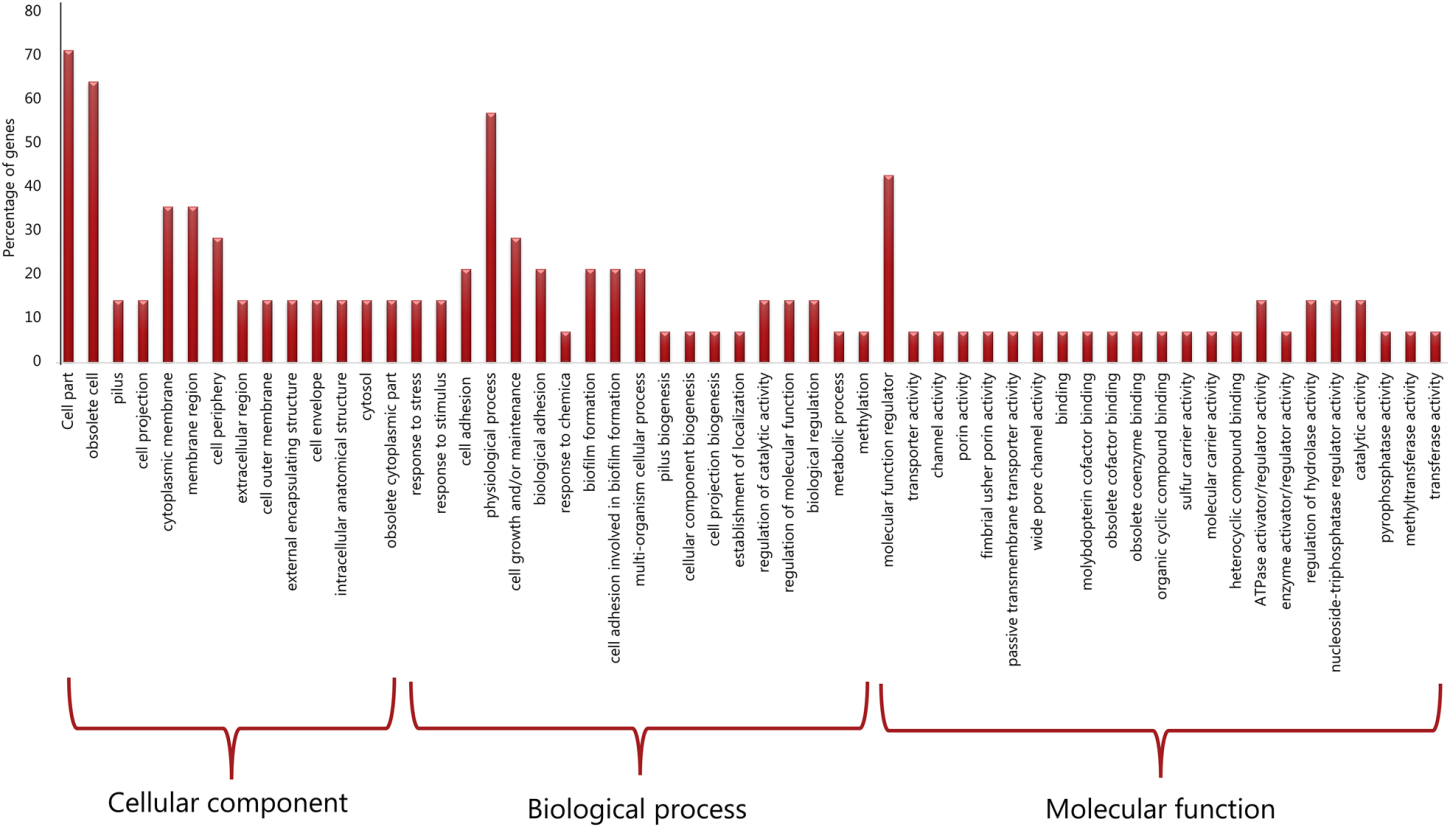
Gene oncology enrichment depicted from Part C representing unique CDS for *S. enterica* subsp. *salamae* serovar 42:r-.

A much closer look into each of the observed unique parts in the accessory genomes unique to the serovars was done with a GO annotation analysis and revealed that the functional GO-terms rather point to a variety of gene enrichment in the biological processes and molecular functions. A specific look into Part C reveals a low level of gene enrichment in the pathways of biofilm formation, response to stress and stimulus, which indicates limited environmental adaptation and pathogenicity (Fig. 4, portion biological process).

### Genetic variations in our strains within *Salmonella enterica* genealogy

**Figure 5:**
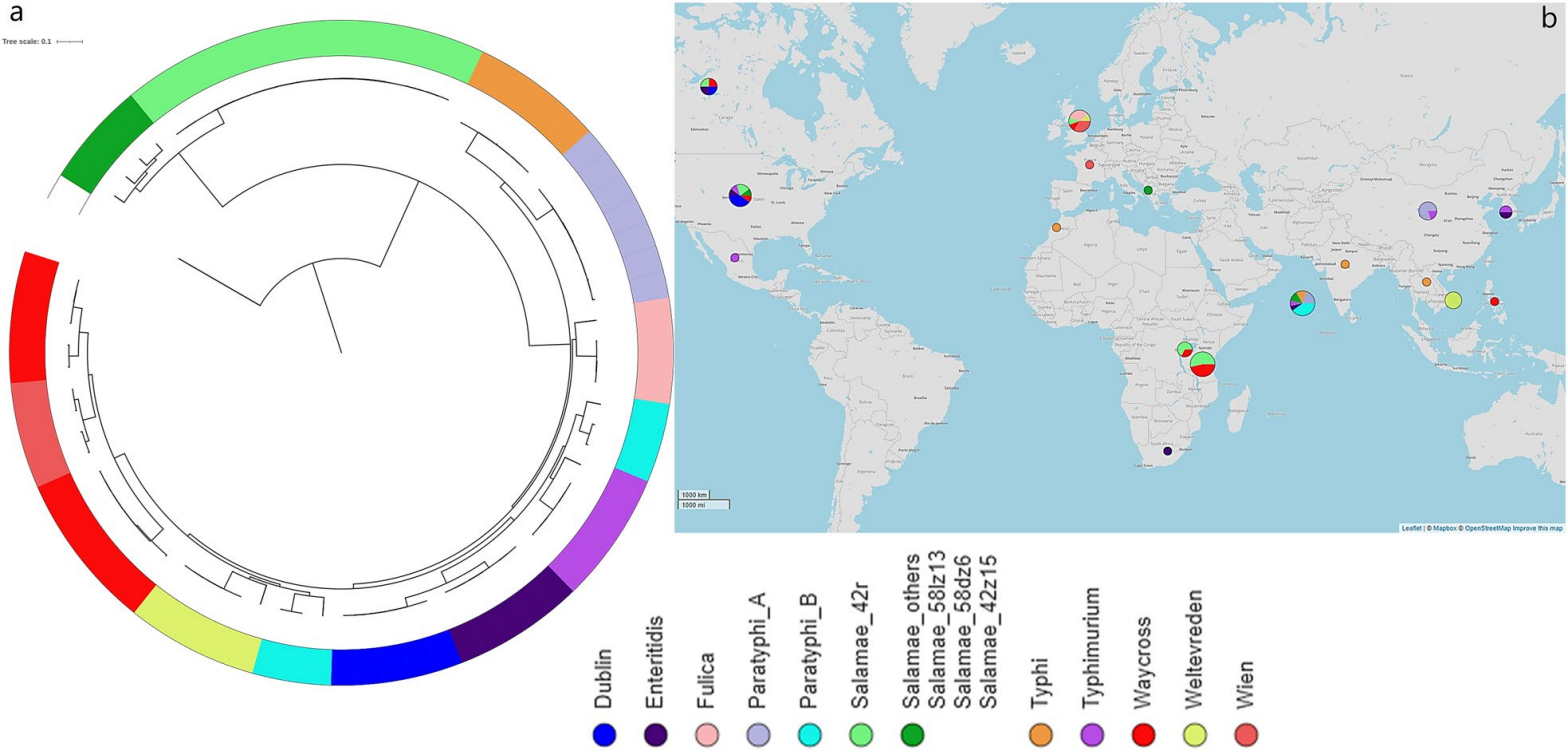
Phylogeny of *S.* Waycross and *S.* Salamae from Lake Victoria in a global *S. enterica* context; (**a**) SNP-tree showing clustering of the strains colored by serovars; (**b**) geographical distributions of the serovars included in the analysis. Bigger circles illustrate geographical areas with higher number of strains included in the analysis. The Map in panel (**b**) is generated from Microreact (https://microreact.org/) using geographical coordinates of the place of isolation of analyzed strains.

The phylogenetic analysis of gene sequences from 75 *enterica* and non-*enterica* subspecies of *Salmonella* enterica isolated from 1985 to 2018 revealed two large clusters based on subspecies irrespective of the source of isolation i.e. environmental or clinical origin (Fig. 5). This suggests that it is unlikely that *S*. Salamae 42:r- and *S*. Waycross are natural occurring aquatic microorganisms as they showed wide-genetic variations as compared with for instance *S*. Weltevreden, which frequently are isolated from the aquatic environment. The analysis also showed a further convergence of *Salmonella* strains by serovars as shown in the sub-clades on the tree (Fig. 5).

### Invasion of intestinal epithelial cells

**Figure 6:**
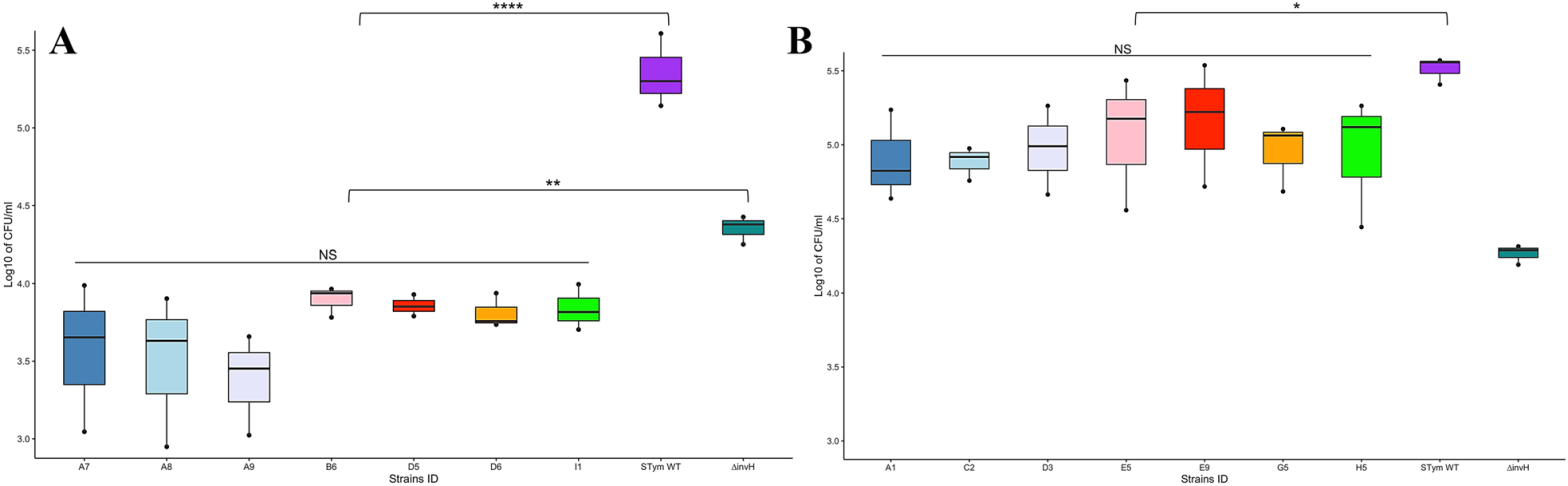
Invasion of *S.* Salamae (**A**) and *S*. Waycross (**B**) in human intestinal epithelial cell line INT-407. Each box plot represents one strain with values from three biological replicates and two technical replicates at each round. NS: not significant; *p* > 0.05; *p < 0.05; **p < 0.01,****p < 0.0001.

The log distribution of bacteria counts for the three experimental replicates from inoculum, adhesion and invasion studies are shown in Fig. S3 and Fig. S4, respectively for *S*. Salamae and *S*. Waycross. The adhesion to the INT-407 epithelial cells ranged from 0.01% to 0.06% for *S*. Salamae strains, while 0.23 to 0.43% was recorded as adhesion rates for the wild type *S*. Typhimurium (Table S3). The cell invasion analysis shows that within *S*. Salamae tested strains, the number of bacteria that invaded epithelial cells varied non-significantly from 1.6 × 10^3^ to 9.8 × 10^3^ CFU/mL (*p* = 0.3). However, when compared to the wild type *S*. Typhimurium (1.39 × 10^5^ to 4.06 × 10^5^ CFU/mL), the Salamae serovar strains show significantly negligible invasion rates (*p* = 0.000000011) and even the *invH* mutant of *S*. Typhimurium (used as a negative control) (1.78 × 10^4^ to 2.67 × 10^4^) was more invasive in epithelial cells than *S.* Salamae (*p* = 0.004) (Fig. 6A).

On the other hand, the epithelial cell adhesion rates in *S*. Waycross varied from 0.07% to 1.3%; however, there was no significant intra-serovar variation for *S*. Waycross in their cell invasion ability (2.78 × 10^4^ to 2.72 × 10^5^ CFU/mL, *p* = 0. 956). Nevertheless, compared to *S*. Typhimurium wild type (2.56 × 10^5^ to 3.72 × 10^5^ CFU/mL), the *S*. Waycross strains showed a slightly lower cell invasion levels (*p* = 0.014). However, *S*. Waycross remain significantly more invasive than the *invH* mutant of *S*. Typhimurium (*p* = 0.0009) as opposed to *S.* Salamae (Fig. 6B).

### Intracellular survival and replication

**Figure 7:**
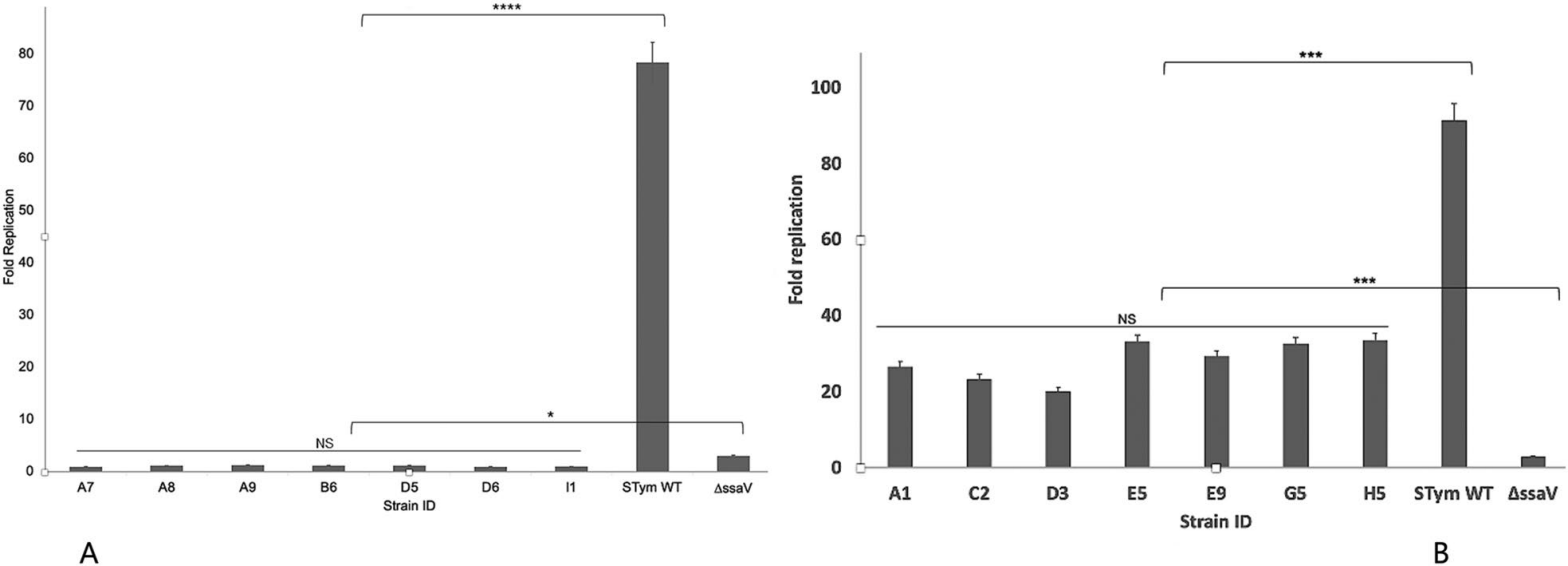
Intracellular replication of *S*. Salamae (**A**) and *S.* Waycross (**B**) in mouse macrophage J774 cell lines. NS: not significant; *p* > 0.05; *p < 0.05; ***p < 0.0001; ****p < 0.00001.

After the J774 macrophages phagocytized the experimental bacterial strains, we recorded 0.17–0.9% as intra-cellular survival rate among *S*. Salamae 42-r strains. However, these could not replicate to higher number inside macrophages after 18 h post infection as their fold replication ranged between 0.22% and 3.2% (Table S3). This explains the significant difference when compared to the intracellular fold replication rate of wild type *S* Typhimurium that varied from 77.5% to 80% (*p* = 3.02 × 10^–34^, Fig. 7A). Nevertheless, of 0.26–1.6% of *S.* Waycross strains that were obtained during intracellular survival, up to 18.6–45.9% were able to replicate within the macrophages. The *S*. Waycross strains showed a significantly higher intracellular replication compared to *ssaV* mutant of *S.* Typhimurium, whose fold replication rates varied between 2.4% to 3.8% (*p* = 3.57 × 10^–4^). The fold replication rates observed in *S*. Waycross were however not as high as the wild type positive control (86.2–97.5%, Table S3, Fig. 7B).

## Discussion

We isolated two rare serovars *S.* Salamae 42:r- ST 1208 and *S*. Waycross ST 2460 and ST 3691 in Nile perch and water samples collected from deep offshore waters in Lake Victoria in Tanzania^11^. The presence of *Salmonella* in Nile Perch is often the cause of restrictions on export of the fish products into the international markets. Recent reports of Nile perch products from Tanzania exported to EU member states such products were rejected or detained due to contamination by *Salmonella* spp.^11^. *Salmonella* spp. have been found in fresh and processed Nile perch from Lake Victoria in Kenya^3,12^. Existing evidence from the global epidemiology of *Salmonella* however suggests that only serovars within the subspecies enterica of *Salmonella enterica* are implicated in human salmonellosis^9^. It is therefore important to determine the subspecies of *Salmonella* strains isolated in seafood otherwise rejections of fish/fish products containing *Salmonella* spp. could result in unnecessary food waste and economy loss.

Existing literature have shown that *Salmonella Salamae serovar Sofia* were able of colonizing chicken organs but could not cause symptomatic disease^17^. Other studies have also reported that non-enterica subspecies such as subspecies *arizonae* and *diarizonae* show significant reduction of intestinal colonization, persistence and systemic spread in murine models with subsequent decrease in faecal shedding^18^. These characteristics document avirulent features of the non-enterica subspecies. The characterization of non-enterica *Salmonella* strains at subspecies level is therefore important to determine their pathogenic potential. The genomic analysis of our strains revealed only the presence of the *aac(6’)laa* gene encoding aminoglycoside resistance and a mutation in *parC* (p.T57S) known to be associated with nalidixic acid resistance observed in some strains^11^. A few isolates showing phenotypic resistance to sulfamethoxazole (Table 1) were not found to contain any encoding resistance gene, a discrepancy that is increasingly reported in many bacteria^10^. Overall, our strains were susceptible to most antimicrobials tested. This suggests that the strains may not have been exposed to antimicrobials, which is further supported by the fact that they were isolated in off-shore lake waters containing low levels of fecal contamination^11^. Knowing that bacteria can pick-up genetic material from each other, it is possible that, when in the same niche, epidemic strains could transfer virulence or antimicrobial resistant genes to non-pathogenic strains through their mobile genetic elements.

The pathogenicity of *Salmonella* is mainly dependent upon the two *Salmonella* encoded T3SSs (Type III Secretion systems), i.e. SPI-1 and SPI-2, which are required for different stages of salmonellosis, namely cell invasion and intracellular replication^19,20^. SPI-1 and SPI-2 were present in our strains of both *S.* Salamae and in *S.* Waycross. However, the significant deletions recorded in the two pathogenicity islands (Fig. 1) especially in *S.* Salamae corroborates existing literature that this subspecies lacks key virulence genes in these pathogenicity islands required causing human salmonellosis^21^. With cell invasion assays, we further confirmed the low pathogenicity of the *S.* Salamae strains as they could not invade the INT-407 epithelial cells, not even close to the levels observed in the *invH* mutant of *S.* Typhimurium 4/74. In fact, all the *S.* Salamae strains lacked the *invH* gene that has been knocked-out in the negative control, confirming the role of these set of *inv* genes in the invasion ability of *Salmonella*^13^. While the tested *S*. Waycross strains, as an enterica subspecies seem to invade the epithelial cells at increased levels compared to *S.* Salamae, the wild type *S.* Typhimurium 4/74 were significantly more invasive.

The deletions observed mainly in the SPI-2 encoding genes of *S.* Salamae explain why they are not able to survive and replicate within the murine J774 macrophages. Studies have shown that non-*enterica* subspecies serovars including *S.* Salamae do not internalize well and do not replicate in macrophages and also demonstrate a severely reduced intestinal colonization and intestinal persistence^13,21^. Such inabilities are attributable to the deletions of important genes that are found in the subspecies *enterica*, explaining why *S*. Waycross strains showed higher intracellular replication rates although not as high as the wild type *S.* Typhimurium.

Apart from the T3SSs, the ability to form biofilm has been documented as important factor for environmental persistence and virulence in *Salmonella* as it favours the survival of the bacteria under harsh conditions including low-nutrient conditions, acidic pH, and varying temperatures^22^; all factors increasing ability to infect a host. In this context, both *S.* Waycross and *S.* Salamae contained few genes associated with biofilm formation suggesting a potentially reduced ability to form biofilm, a feature that could impede their overall long term environmental adaptation as opposed to *S*. Weltevreden^10^.

While our characterizations of the *S.* Waycross isolated from fish and water documented some pathogenic potential, the findings show that *S.* Salamae should not be regarded as an important human pathogen. It is proposed that *Salmonella* spp. isolated from seafood should be further characterized to determine their pathogenic potential. Such characterizations are currently not done by most food safety laboratories because the existing food safety legislations still consider all sub-species and serovars of the genus *Salmonella* pathogenic to humans. Our data together with existing literature supports that not all *Salmonella* subspecies and serovars are pathogenic, and that efforts should be made towards revisiting legislations and equipping food safety laboratories with knowledge and analytical methods to ensure less waste of food while still ensuring food safety. Currently most laboratories responsible for microbiological food safety analysis and issuing export certifications do not perform whole genome sequencing. Therefore, based on the outcomes of the accessory genomes studied a more practical suggestion would be to design subspecies-specific primers targeting for instance unique CDS on SPI-1 and SPI-2 genes for PCR assays that can be performed at any laboratory to distinguish the non-enterica subspecies. Overall, the outcomes of this study provide useful background information, especially for public health authorities and researchers that can found the basis for designing and implementing further investigations that address the limitations and add further perspectives to the present study.

## Materials and methods

### Genomic analysis

#### *Salmonella* isolates, DNA extraction and whole genome sequencing

*Salmonella enterica* subsp. *enterica* serovar Waycross and *S. enterica* subsp. Salamae serovar 42:r:- were previously isolated from Nile perch (*Lates niloticus*) and water samples collected far offshore in Lake Victoria in waters with low levels of fecal contamination; details on sampling strategy are in the previous study^11^. The isolates were originally serotyped based on the White-Kauffmann-Le Minor (WKL) scheme^23^, and tested for susceptibility to 14 antimicrobial agents by the MIC method^11^. All seven *S*. Salamae and seven of the *S*. Waycross isolated in that study were selected for whole genome sequencing. Selection of the *S.* Waycross strains was based on differences in sampling dates, sample types and MIC results. DNA was extracted from exponential bacterial cultures using the Maxwell RSC culture cell’s DNA kit following the manufacturer’s protocol and the automated Maxwell RSC machine (Promega, Wisconsin, USA). The complete genomes were sequenced with the MiSeq instrument (Illumina, Inc, San Diego, CA, USA). The sequence reads were submitted to the European Nucleotide Archive under the project accession number PRJEB34642.

### Read processing and assembly

Raw sequence reads were trimmed with bbduk2^24^, using the score cut-off of 20 and the reads quality was evaluated with FastQC v0.11.5 before and after quality check. Trimmed reads were assembled with Spades v3.13.0^25^ using error correction, coverage cut-off = 2 and the kmer sizes 21, 33, 55, 77, 99 and 127. Contigs shorter than 200 bases were discarded and the quality of the de novo assembled contigs was analysed using Quast (v4.5)^26^.

### Characterization of the genomes

A serovar prediction analysis for confirmation of the two serovars was performed applying *Salmonella in-silico* Typing Resource (SISTR)^27^. MLST was determined based on the Achtman seven housekeeping genes MLST scheme from Enterobase^28^. General characteristics of the assembled genomes were determined using tools available at Center for Genomic Epidemiology (https://cge.cbs.dtu.dk/services/). These included SPIFinder v1.0 for the detection of *Salmonella* Pathogenicity Islands (SPIs), PlasmidFinder v2.0^29^, and ResFinder v4 (using Abricate) for the detection of antimicrobial resistance genes^30^ in comparison with the phenotypic MIC microdilution test initially reported^11^. The *in-silico* analyses of the genomes also included identification of prophages using PHASTHER^31^ to detect and compare prophage insertions within the genomes of each strains.

### Comparative genomics of virulence determinants

Pathogenic markers encoded in the main *Salmonella* Pathogenicity Islands (SPIs) were first determined using the Pathogenicity Islands Database PAIDB v.2.0^32^, and then the VF*analyser* of the Virulence finder database (http://www.mgc.ac.cn/cgi-bin/VFs/v5/main.cgi?func=VFanalyzer) was used to further characterize virulence determinants and compare them with the *S*. Waycross and *S*. Salamae genomes. Genes located on the SPIs were further investigated one-by-one by local BLAST search against our genomes with low thresholds set at 50% query cover and 50% percent ID to avoid eventual “false negative” gene absence outcomes. Moreover, the detected genes on the SPIs were confirmed in Artemis^33^ by the absence of premature stop codons in the annotation.

A pangenome analysis was performed to describe the specificities of the genomes of 75 *Salmonella* strains isolated from humans, animals, food and the environment obtained from different countries from 1985 to 2018 (up to the isolation date of our strains) (Table S4). These include *S*. Typhi, *S*. Typhimurium, *S*. Paratyphi A and B, *S*. Wien, *S*. Waycross, *S*. Salamae, *S.* Dublin, *S*. Enteritidis and *S*. Weltevreden. All genomes were annotated using Prokka^34^ with the annotated GFF3 files used as an input to the Roary (v.3.7.0)^35^ pangenome analysis tool in a Linux interface. We then used the binary presence/absence data of the accessory genome produced in Roary to calculate the associations between all genes in the accessory genome and the source types, as well as serovars of the identified isolates by employing Scoary v.1.6.11^36^.

The genes presence/absence in the pangenome along with the accessory genome was visualized in Phandango^37^. The unique coding sequence (CDS) blocks observed per subspecies and/or serovar in Phandango were extracted. We then applied the ‘*query_pan_genome*’ function of Roary to retract them as multi-fasta files. Using a blast atlas analysis from the GView server (https://server.gview.ca), the block of CDS that was unique to the enterica subspecies where *S*. Waycross belonged, was mapped back to the reference *S*. Waycross genome SAMN04160804. The same analysis was done for the unique CDS of all *S*. Salamae using the reference SAMEA2665118 and more specifically for the serovar *S*. Salamae 42:r- using the reference genome SAMN10638893.

To identify functional roles of the CDS unique to each subspecies and/or serovar we performed a functional gene ontology annotation of the targeted CDS with eggNOG-mapper^38^. This tool performs annotation with similar precision as the widely used homology-based approaches: BLAST and InterProScan, but runs about 15 × faster than BLAST and at least 2.5 × faster than InterProScan^38^. The obtained functional profiles based on GO-terms were classified in biological and molecular functions and reported for each GO-annotated gene of the unique CDS to determine pathways related to pathogenicity.

To understand the role of the targeted unique CDS in each subspecies and/or serovar, the extracted multi-fasta files were used as input and analysed with the VRprofile^39^ server that generates rapid information on virulence and antimicrobial resistance determinants within pathogenic bacterial genomes. The resulting data was visualized as graphics using Microsoft excel and the Gview server (https://server.gview.ca/). The unique CDS files were further re-assessed ResFinder, PHASTER and RAST to identify potential virulence, resistance and environmental persistence factors.

### Phylogenetic analysis across enterica and non-enterica subspecies

All 14 sequenced strains along with the public genomes used in the pangenome analysis were included to construct a phylogenetic tree. This aimed to investigate genetic diversities within and between the *Salmonella* serovars mainly to understand how our serovars diverge from the *enterica*-subspecies of *Salmonella enterica*. We used FastTree for SNP calling through CSIphylogeny^40^ where *Salmonella bongori* (accession SAMN02603391) was used as outgroup. The newick file of the tree was visualized in Microreact (https://microreact.org/showcase) to display the spatio-temporal distribution of the strains collections along with the SNP-tree. The pairwise SNPs data are shown in the in supplementary Table S5.

### Comparative genomics for identification of environmental adaptation markers

Since the strains were isolated from fish and water collected at deep waters with low levels of fecal contamination^11^, a function-based comparison was made between *S*. Waycross and *S*. Salamae and the aquatic bacterium *Vibrio cholerae* O1 strain N16961 based on the comparison of the metabolic reconstruction from the RAST server^41^ that allows to compare the functioning parts of two organisms.

### Cell infection studies

#### Bacterial strains and cell lines

All *S*. Salamae 42:r- and *S*. Waycross strains analyzed by WGS were included in experimental cell infection studies. The reference strain *S*. Typhimurium 4/74 was used as positive control in all experiments. The isogenic mutant for *invH* of *S.* Typhimurium 4/74 served as negative control for the epithelial cells infection. This strain has a mutation in the *invH* gene, reducing its rate of invasion compared to the wild type. *S*. Typhimurium 4/74 Δ*ssaV* was used as negative control for the intracellular macrophage infection study. This strain is deficient in *ssaV*, a structural component of the SPI2-encoded T3SS reducing its rate of intracellular replication with regard to the wild type. The wild type and mutants were described in a previous study^42^.

The human embryonic intestinal epithelial cell line INT-407 (HeLa-derived epithelial cells) served to assess the invasion ability of *S*. Salamae and *S*. Waycross in comparison to the control strains. On the other hand, intracellular survival and replication within macrophages was investigated for our strains using the mouse monocyte-derived macrophage cells J774.

### Infection of epithelial cell lines

The epithelial cells were cultivated in DMEM (Gibco) supplemented with 10% (v/v) heat-inactivated fetal bovine serum (FBS, Fertile Bovine Serum, Invitrogen) and 25 µg/mL gentamicin to prevent bacterial contamination. The cells were grown in a humidified 37 °C and 5% CO_2_ incubator. Twenty-four hours prior to infection, the INT-407 cells (Cell Lines Service (CLS), Heidelberg, Germany) were seeded in two 24-well plates (one plate for determination of adhesion and the other for invasion) in a concentration of 2.5 × 10^5^ cells per mL. The bacteria (experimental strains as well as controls) were grown for 16 h at 5 rcf and 37 °C in Luria Broth (Difco, Maryland, USA). Bacterial cultures were centrifuged at 8,228 rcf for 8 min. The suspensions were adjusted to OD_600_ = 0.25 (2.5 × 10^8^ bacteria/ml) in 0.9% NaCl and added to monolayers of the eukaryotic cells in both 24-well plates (labeled T1 and T2) at a multiplicity of infection of 100:1 (bacteria to eukaryotic cell) without antibiotic. Inoculum counts were verified by plating aliquots of the inoculated suspension on LB agar plates (counts before infection). After 30 min of infection at 37 °C, 5% CO_2_, the media from both plates was removed and monolayers were washed twice with 0.9% NaCl. At this point, T1 plates are processed straight away for plating while fresh media containing 100 µg/mL gentamicin was added to T2 plates to kill extracellular bacteria then they were incubated for further 2 h at 37 °C, 5% CO_2_. To enumerate adhered (T1 = 30 min) and invaded bacteria (T2 = 2 h), cells were washed twice with 0.9% NaCl and lysed in 1 mL 0.1% Triton X-100 (v/v). The viable bacteria were enumerated by colony counts of lysate dilutions plated on LB agar. The experiments were performed in triplicates for biological replication with two technical replicates during each round.

### Infection of macrophages

Macrophage cell lines J774.1 (Cell Lines Service (CLS), Heidelberg, Germany) were cultured in RPMI (Gibco) supplemented with 10% (v/v) heat-inactivated FBS and 25 µg/mL gentamicin. Cells were incubated in a humidified 37 °C, 5% CO_2_ incubator. The bacteria were grown in LB to a stationary phase, and harvested at 8,228 rcf for 5 min and resuspended in 0.9% (w/v) NaCl. Following similar procedures as in the epithelial cell infection, bacteria were added to eukaryotic cells at a multiplicity of infection of 10:1 (bacteria/macrophage). The monolayer cells with bacteria were centrifuged at 12 rcf for 3 min immediately after addition of the bacteria followed by incubation for 25 min at 37 °C, 5% CO_2_ without antibiotic. Enumerations of the bacteria in the inoculum were verified by plating onto LB agar plates (counted before infection). After 30 min, the media was removed and monolayers were washed twice with 0.9% NaCl. At this time point (corresponding to time 0 for phagocytosis) fresh RPMI containing 10% heat-inactivated FBS and 100 µg/mL gentamycin was added to kill the extracellular bacteria and the plates were incubated for 1 h (T1) at 37 °C in 5% CO_2_ to assess intracellular survival. Thereafter, cells in replication plates were washed twice with 0.9% NaCl and incubated with RPMI containing 10% heat-inactivated FBS and 25 µg/mL gentamycin for 16 h (T2). For enumeration of bacteria, eukaryotic cells were washed twice with 0.9% NaCl, subsequently lysed in 0.1% Triton X-100 (v/v). The viable intracellular bacteria were enumerated by colony counts of lysate dilutions plated on LB agar plates. For intracellular survival rates, bacteria were enumerated at t = 1 h (1 h post-uptake or post-phagocytosis), and for intracellular replication, bacteria were counted at t = 16 h (~ 18 h post-infection). The experiments were performed in triplicates for biological replication with two technical replicates during each round.

### Statistical analysis

In the epithelial cell experiments, rates of adhesion and invasion are expressed as the CFU/mL after 30 min and 2 h post infection with respect to the initial inoculum (Table S3). In the macrophage infections, the intracellular survival was determined as the CFU/mL at T1 with respect to the initial inoculum, while the fold replication was calculated as the ratio of bacteria recovered from host cells at T2 and T1 (Replication/Survival). Statistical significance of the differences between the strains was determined using R and RStudio 1.1.1717 package, where comparison within our experimental strains was performed using one-way ANOVA and comparisons between experimental strains and controls was performed using the pairwise t test comparison. The Bonferroni adjusted *p* value was used for significance.

### Ethics statement

The present study required no ethical approval because as indicated in the Materials and Methods, this study only analyzed archived bacterial strains initially isolated by a previous study^11^. All methods were carried out in accordance with relevant guidelines and regulations.

## Supplementary Information

Supplementary Figure S1.

Supplementary Figure S2.

Supplementary Figure S3.

Supplementary Figure S4.

Supplementary Table S1.

Supplementary Table S2.

Supplementary Table S3.

Supplementary Table S4.

Supplementary Table S5.
