# Supplementary figures and images for: Salmonella Salamae and S. Waycross isolated from Nile perch in Lake Victoria show limited human pathogenic potential
Source: Sci Rep. 2022 Mar 10;12:4229. doi: 10.1038/s41598-022-08200-5 (PMC8913728; doi:10.1038/s41598-022-08200-5)

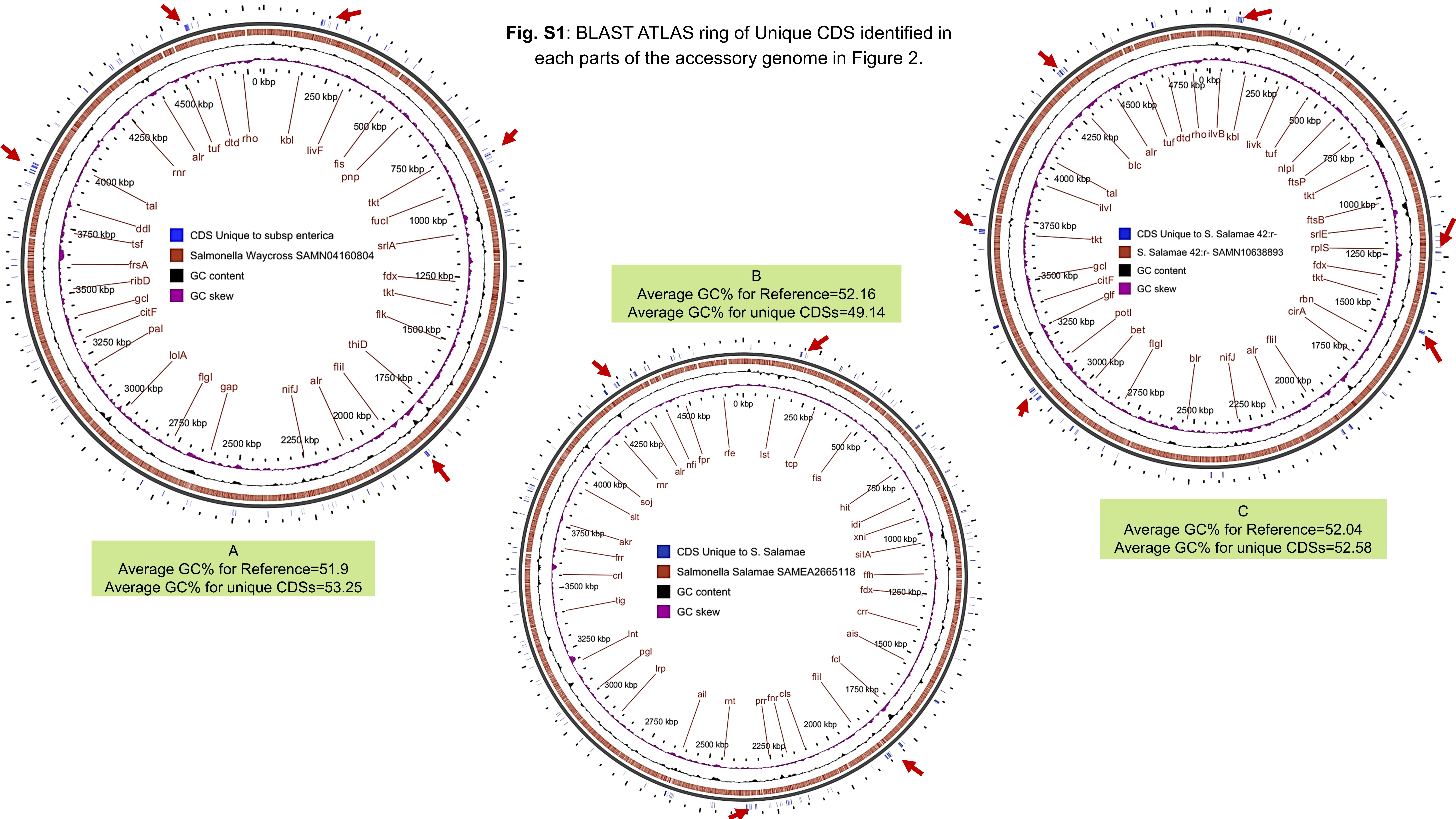

Supplement: Supplementary file 1 — Supplementary Figure S1. [file 41598_2022_8200_MOESM1_ESM.jpg]

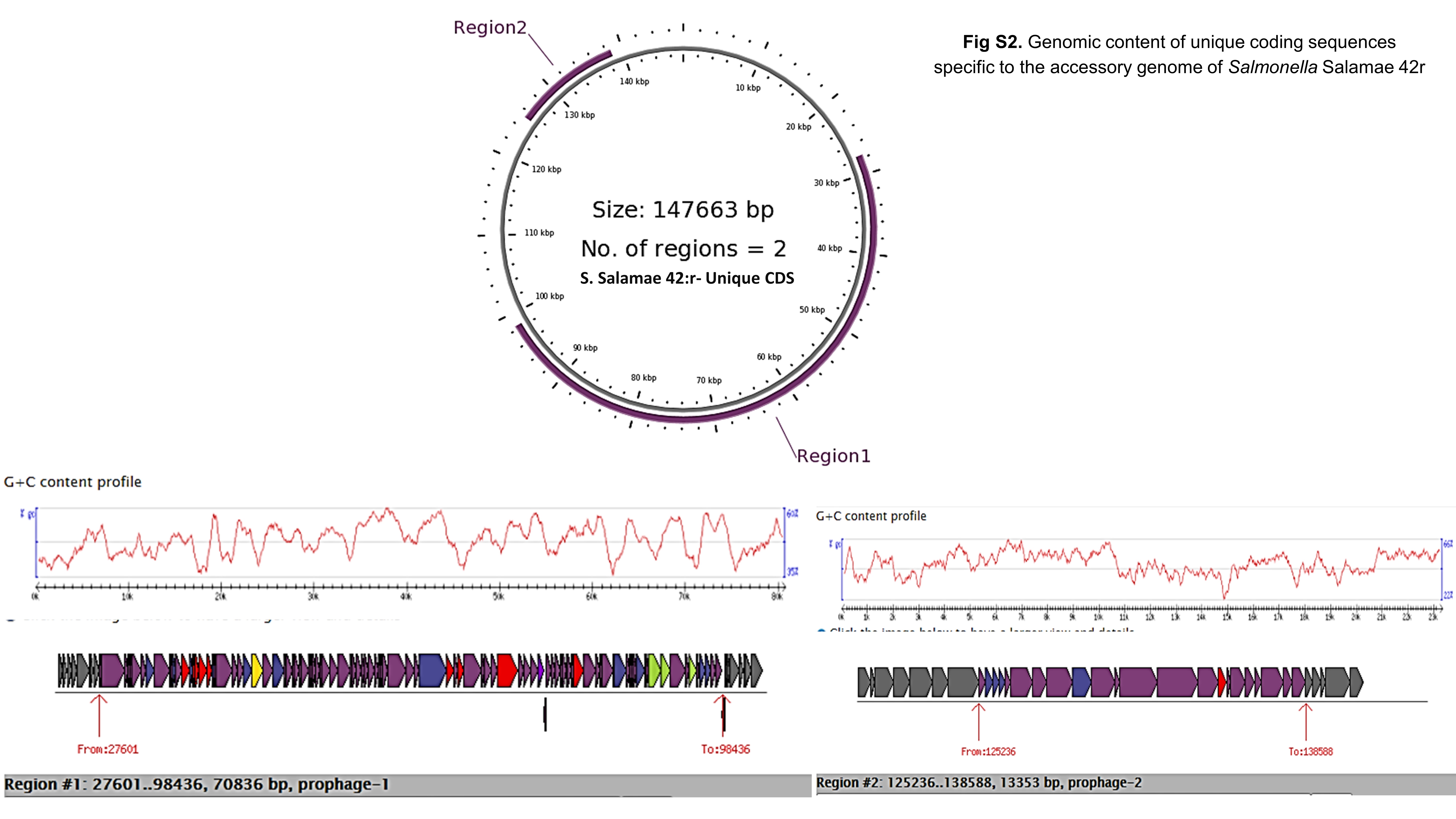

Supplement: Supplementary file 2 — Supplementary Figure S2. [file 41598_2022_8200_MOESM2_ESM.jpg]

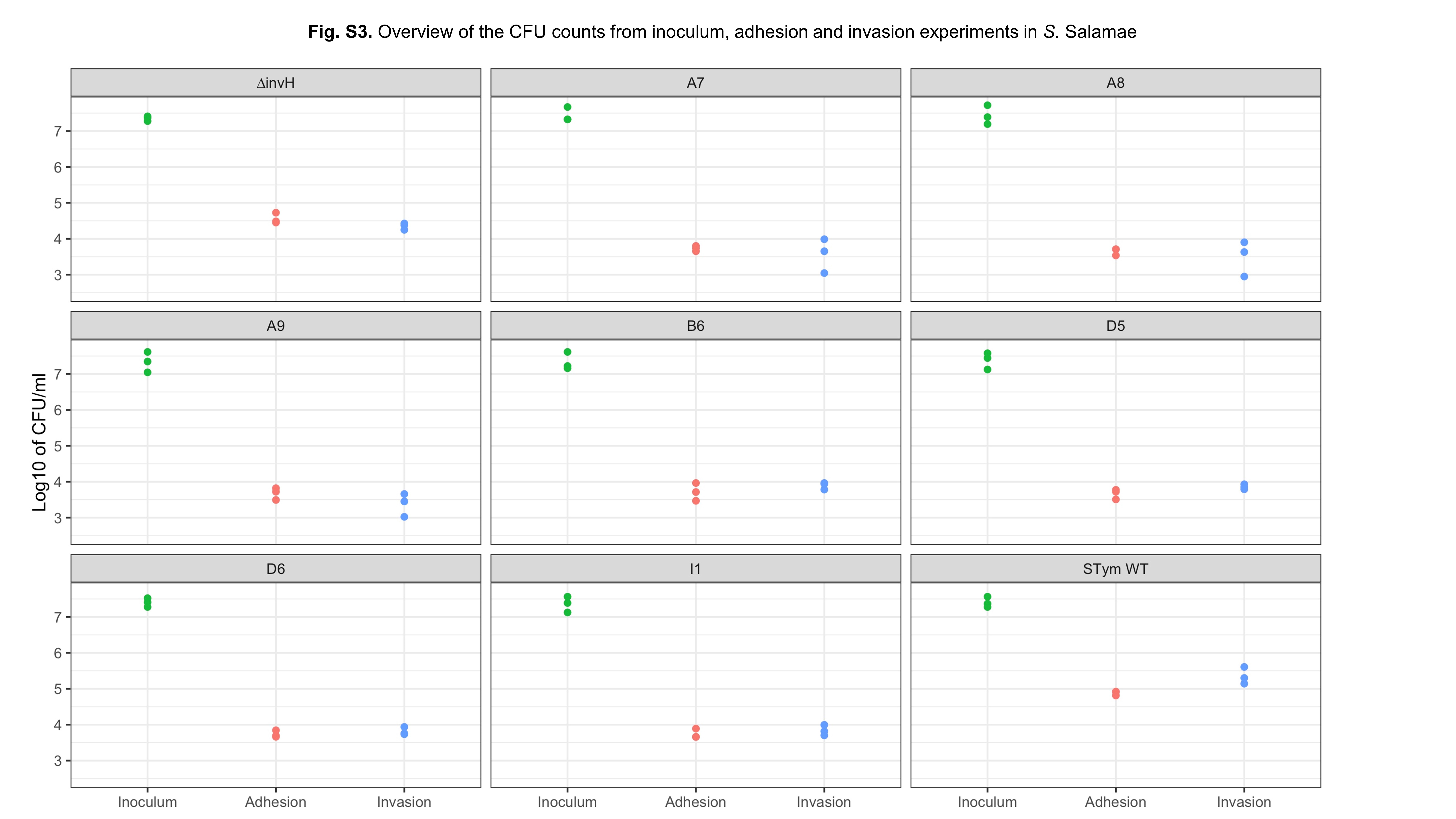

Supplement: Supplementary file 3 — Supplementary Figure S3. [file 41598_2022_8200_MOESM3_ESM.jpg]

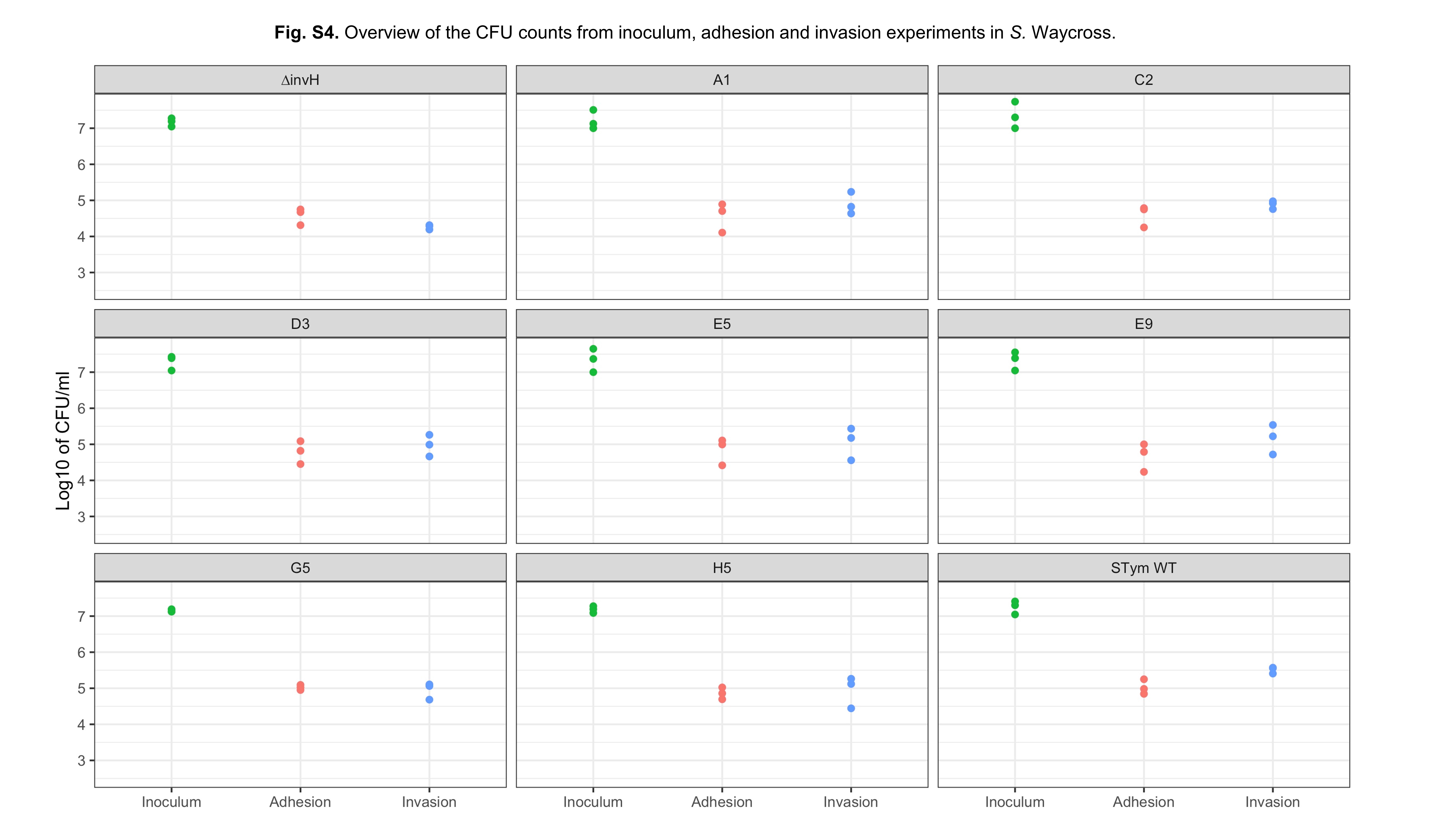

Supplement: Supplementary file 4 — Supplementary Figure S4. [file 41598_2022_8200_MOESM4_ESM.jpg]
